# Tick-borne Encephalitis Vaccine Failures: A 10-year Retrospective Study Supporting the Rationale for Adding an Extra Priming Dose in Individuals Starting at Age 50 Years

**DOI:** 10.1093/cid/ciz176

**Published:** 2019-03-07

**Authors:** Karin E Hansson, Anja Rosdahl, Mona Insulander, Sirkka Vene, Lars Lindquist, Sara Gredmark-Russ, Helena H Askling

**Affiliations:** 1 Center for Infectious Medicine, Department of Medicine Huddinge, Karolinska Institutet, Karolinska University Hospital, Stockholm, Sweden; 2 Department of Infectious Diseases, Södersjukhuset, Stockholm, Sweden; 3 School of Medical Sciences, Örebro University, Sweden; 4 Department of Infectious Diseases, Örebro University Hospital, Sweden; 5 Department of Communicable Disease Control and Prevention, Stockholm County, Sweden; 6 Public Health Agency of Sweden, Solna, Sweden; 7 Department of Medicine, Karolinska Institutet, Huddinge, Sweden; 8 Department of Infectious diseases, Karolinska University Hospital, Sweden; 9 Division of Infectious Diseases, Unit for Infectious Diseases, Karolinska Institutet, Stockholm, Sweden; 10 Department of Communicable Disease Control and Prevention, Sörmland County, Sweden

**Keywords:** TBE, TBEV, vaccination, vaccine failure

## Abstract

**Background:**

Southern Sweden is endemic for tick-borne encephalitis (TBE), with Stockholm County as one of the high-risk areas. Our aim in this study was to describe cases of vaccine failures and to optimize future vaccination recommendations.

**Methods:**

Patients with TBE were identified in the notification database at the Department of Communicable Disease Control and Prevention in Stockholm County during 2006–2015. Vaccine failure was defined as TBE despite adherence to the recommended vaccination schedule with at least 2 doses. Clinical data were extracted from medical records.

**Results:**

A total of 1004 TBE cases were identified, 53 (5%) were defined as vaccine failures. In this latter group, the median age was 62 years (6–83). Forty-three (81%) patients were aged >50 years and 2 were children. Approximately half of the patients had comorbidities, with diseases affecting the immune system accounting for 26% of all cases. Vaccine failures following the third or fourth vaccine dose accounted for 36 (68%) of the patients. Severe and moderate TBE disease affected 81% of the cases.

**Conclusions:**

To our knowledge, this is the largest documented cohort of TBE vaccine failures. Vaccine failure after 5 TBE vaccine doses is rare. Our data provide rationale for adding an extra priming dose to those aged ≥50 years.

## Teaser

This 10-year retrospective study includes 53 patients (median age 62 years, 6-83) with TBE vaccination failure. Forty-four (81%) patients were aged ≥50 years. Based on our data, we propose adding an extra priming dose for individuals aged ≥50 years.

Tick-borne encephalitis (TBE) is one of the most important causes of viral encephalitis in Europe, with 5000–10 000 reported cases annually [1, 2]. TBE is endemic in 27 European countries [3], with the highest annual incidence rate (5–18.6/100 000) in the Czech Republic, the Baltic countries, and Slovenia [4]. TBE virus is a flavivirus, and the hard tick *Ixodes ricinus* is the principal vector for the European TBE virus subtype, the main cause of European TBE cases [1, 2].

Typically, TBE caused by the European subtype is a biphasic disease in adults, with an initial episode of fever, fatigue, myalgia, and headache. After a symptom-free interval, a second phase develops with a broad spectrum of neurological symptoms, ranging from mild meningitis to severe meningoencephalitis or meningoencephalomyelitis [5–7]. Whereas the mortality is low (<2%) [6, 8, 9], persisting neurological sequelae have been reported in 31%–40% of patients [5, 7] and a self-reported perception of cognitive impairment is common even after several years [6, 10].

There is no specific treatment for TBE, but the disease can be prevented by active immunization [11]. Two inactivated vaccines are presently registered in Europe: FSME-immune (Pfizer) and Encepur (GlaxoSmithKline), and they are considered interchangeable [12]. Although there are no controlled trials to show protective efficacy, field studies in Austria, where approximately 85% of the population have received at least 1 dose of TBE vaccine, have shown 95%–99% effectiveness [11, 13]. However, an inferior vaccine response, measured by antibody titers or neutralization tests, has been shown with increasing age and immunosuppressive comorbidities [14–17]. Vaccine failures have been described in 1%–6% of all TBE cases [8, 9, 17–31]. Most publications are case reports, and the definition of a vaccine failure is not consistent, thus making data difficult to compare.

In Sweden, notification of TBE is mandatory, and the reporting rate of laboratory-verified disease is complete. Stockholm County is a high-incidence area despite a 50% vaccination coverage [32]. The standard vaccine schedule is 2 primary doses (0, 1–2 months) followed by additional doses at 5–12 months and 3 years. Since 1998, further doses have been recommended every 5 years regardless of age [33]. Also, based on earlier Swedish data that indicate a high proportion of TBE vaccine failures in older age groups [18], an extra priming dose at month 3 was added in the national recommendations in 2010 for all individuals aged ≥60 years.

To optimize TBE vaccination in a high-risk area with TBE-associated morbidity, knowledge about TBE disease despite adherence to the recommended schedule is essential. Our aim in this study was to obtain data of the clinical characteristics and documentation of vaccine doses in patients with TBE vaccine failures in order to identify possible improvements for future vaccination recommendations.

## METHODS

### Study Design and Population

We conducted a retrospective study of all reported TBE cases in Stockholm County during 2006–2015. Cases were identified in the national notification database (SmiNet) of the regional Department of Communicable Disease Control and Prevention. The study was approved by the Regional Ethical Review Board in Stockholm.

### Case Definition of TBE

TBE was defined according to the national notification definition [34] and in agreement with the European Centre for Disease Prevention and Control’s criteria [35] as symptoms and/or signs of meningitis or meningoencephalitis and 1 of the following laboratory findings: TBE-specific immunoglobulin M (IgM) and immunoglobulin G (IgG) in sera or specific IgM in the cerebrospinal fluid, seroconversion in paired sera over time, or detection of TBE-RNA in a clinical specimen.

### Definition of TBE Vaccination Failure

**Figure 1.**
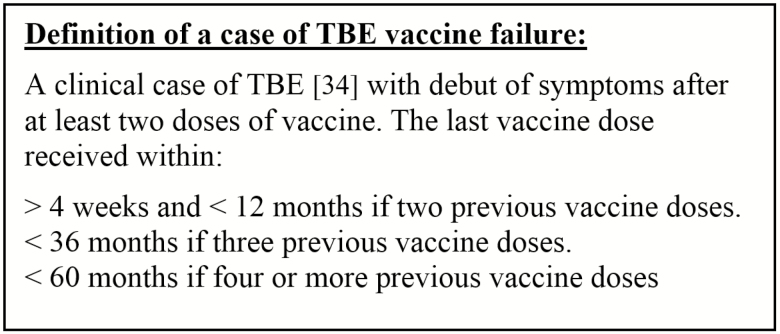
Definition of a case of tick-borne encephalitis (TBE) vaccine failure.

Patients diagnosed with TBE who had previously been vaccinated according to the recommended vaccine schedule with at least 2 doses of vaccine were defined as vaccine failures (Figure 1). A prolonged interval between vaccine doses was accepted as long as the last dose was given within the recommended time in relation to when the patient became ill. Patients were excluded if they became ill with TBE within 1 month after the second vaccine dose due to a potential risk of exposure to TBE virus before seroprotection was achieved.

### Case Data

Base characteristics of age, gender, number of vaccine doses, and the corresponding dates for each dose were collected from the notification database and, if needed, complemented by phone interviews. Clinical and laboratory data were extracted from the patients’ medical records. An assessment of the severity of clinical disease in the acute phase was performed, and patients were classified as having mild, moderate, or severe disease, according to earlier studies [7, 10, 19]. Cognitive dysfunction was defined and evaluated as inability to concentrate, memory disturbances and/or learning difficulties, fatigue, and/or inability to perform executive functions based on the Encephalitis Support Group Questionnaire [10]. Emotional instability was defined and evaluated as mood instability, anxiety, frustration, anger, or signs of depression. Radiculitis was defined as any report of radiating pain following any spinal nerve distribution. All of the described clinical data were defined and searched for by the same coauthor in the medical records at 4 predefined periods following the first signs of central nervous system symptoms: the acute phase at 0–2 weeks, 2 weeks–3 months, 3–6 months and 6–12 months.

### Microbiological Methods

An enzyme-linked immunosorbent assay (ELISA) for TBE IgM and IgG antibodies in serum had been performed in all patients by the hospitals’ regional laboratories. Additional ELISAs and neutralization tests were performed at the Public Health Agency for 51 and 49 patients, respectively, as previously described [18].

## RESULTS

### Base Characteristics

**Figure 2.**
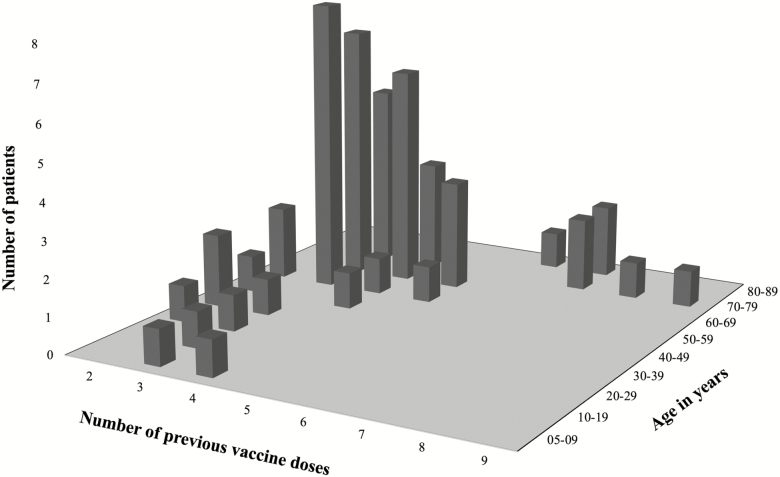
The number of patients who became ill with tick-borne encephalitis regardless of the previous adequate vaccination in Stockholm County, Sweden, 2006–2015. Patients are plotted based on previous number of vaccine doses and their age at the time of disease.

We identified 1004 TBE cases during 2006–2015; among these, 53 (5%) were defined as vaccine failures. They were predominantly men (64%) and the median age was 62 years (6–83), of which 43 (81%) were aged ≥50 years and 2 were children aged <10 years (Table 1 and Figure 2). Underlying disease of any kind was present in 27 (51%) patients. Diseases involving the immune system, such as rheumatoid arthritis, psoriasis arthritis, inflammatory bowel disease, and malignancies, were present in 14 (26%) patients, 8 of whom were on immunosuppressive therapy.

**Table 1.** Demographic and Basic Clinical Data for Patients With Tick-borne Encephalitis Vaccine Failure in Stockholm County, Sweden, 2006–2015

Characteristic	n (%) (n = 53)	Median (Range)
Age (y)	…	62 (6–83)
Male	34 (64)	…
Number of vaccine doses	…	3 (2–9)
Number of months since last dose	…	23 (<1–50)^a^
Comorbidity	27 (51)	…
Neurological disease	5 (9)	…
Psychiatric disease	2 (4)	…
Cardiovascular disease	12 (23)	…
Hematological malignancy	2 (4)	…
Other malignancies^b,c^	3 (6)	…
Inflammatory disease^c^	10 (19)	…
Diabetes mellitus	4 (8)	…
Immunosuppressive therapy	8 (15)	…
Prednisolone equivalents^d^	2 (4)	…
Methotrexate^e^	5 (9)	…
Other immunosuppressive drugs^f^	4 (8)	…

^a^Four patients became ill less than 1 month after their last dose (dose 3 or 5).

^b^Prostate or breast cancer.

^c^One patient with both rheumatic disease and breast cancer.

^d^Dose 2.5 mg or 5 mg/day.

^e^Dose 10–20 mg/week.

^f^Azatioprine, mesalazine, etanercept, hydroxychloroquine.

### Vaccination Doses

The majority of the patients, 36 (68%; median age, 62 years; range, 6–76), had received 3 or 4 vaccine doses prior to becoming ill, and 11 (21%; median age, 71 years; range, 62–83) had received 5–9 doses (Figure 2). All but 1 of the latter patients started their vaccinations after the age of 50 years. In the age group 50–59 years, 8 cases became ill following 3 previous vaccine doses. Thirty-five (66%) patients started their vaccinations at age ≥50 years (missing data in 4 patients). Fifteen patients started their vaccinations after the age of 60 years. Three of these patients became ill 3–5 years after their fourth dose. None of the patients in our cohort had received an extra priming dose in year 1.

Five of 14 patients with diseases involving the immune system had been vaccinated, with any dose, during immunomodulatory treatment. Two patients did not have any immunosuppressive treatment during the time of vaccination. In 7 patients, the corresponding data were missing.

### Clinical Features of Acute Illness: 0–2 Weeks

The most common acute symptoms of disease were fever and headache, present in 50 (94%) and 43 (81%) patients, respectively. The disease was monophasic in 41 (77%) patients. Ataxia was seen in 38 (72%), dysphasia in 30 (57%), cranial nerve affection in 19 (36%), and spinal paresis in 18 (34%) individuals. Among those with cranial nerve affection, double vision was the most common symptom, described in 13 (25%) individuals; 3 of them also presented with abducens nerve palsy. Other cranial nerve involvement caused symptoms and signs of visual disturbances, facial paresis, loss of hearing, difficulties in swallowing, affection of the voice, and dysarthria. Paresis of the upper extremities was present in 7 (13%) patients. Bilateral or unilateral paresis of the lower extremities was observed in 5 (9%), hemiparesis in 3 (6%), and tetraparesis in 3 (6%) patients. Urinary retention was present in 7 (13%) individuals. In addition, 16 (30%) patients presented with symptoms of radiculitis. In all, 47 (89%) patients required hospitalization and 12 (23%) required intensive care treatment. Severe or moderate disease was seen in 22 (42%) and 21 (40%) cases, respectively (Tables 2 and 3).

**Table 2.** Clinical, Laboratory, and Radiological Parameters of the Acute Phase of Tick-borne Encephalitis in Patients With Vaccine Failure in Stockholm County, Sweden, 2006–2015

Clinical Characteristic	n (%) (n = 53)	Median (Range)
Monophasic course	41 (77)	…
Fever	50 (94)	…
Headache	43 (81)	…
Glasgow coma scale (median, range)	…	14 (3–15)
Severity of disease		
Mild^a^	10 (19)	…
Moderate^b^	21 (40)	…
Severe^c^	22 (42)	…
Cerebrospinal fluid findings		
**Pleocytosis**	50 (100)^d^	…
Mononuclear lymphocyte count, cells/µL	…	57 (3–540)
Polynuclear lymphocyte count, cells/µL	…	3 (0–163)
Albumin, g/L	…	529 (254–1198)
Glucose, mmol/L	…	3.5 (2.6–4.6)
Lactate, mmol/L	…	2.2 (1.6–3.9)
Diagnostics apart from routine serology		
Neutralization test positive	49 (100)^e^	…
Magnetic resonance imaging performed	25 (47)	…
Signs associated with encephalitis	9 (36)^f^	…
Electroencephalogram performed	23 (43)	…
Signs associated with encephalitis	20 (87)^g^	…
Signs associated with epileptic activity	2 (9)^g^	…
Hospitalization	47 (89)	…
Days in hospital	…	11 (0–521)
Treatment in the ICU	12 (23)	…
Days in the ICU	…	6 (1–519)
Assisted ventilation	8 (15)	…
Number of days with artificial ventilation	…	21 (2–609)
Rehabilitation^h^	23 (43)	…
Inpatient	15 (28)	…
Number of days	…	28 (5–245)
Outpatient	14 (26)	…
Number of days	…	71 (7–210)
Mortality	3 (6)	…

Abbreviation: ICU, intensive care unit.

^a^Signs of meningitis and normal electroencephalography when applicable.

^b^Mild signs of altered consciousness with diffuse symptoms such as “slow cerebration,” confusion, and/or focal neurological signs such as ataxia, tremor, and dysphasia.

^c^Severe signs of altered consciousness and/or multifocal neurological symptoms.

^d^Fifty of 53 patients had a lumbar puncture.

^e^Percentage of those where neutralization test was performed.

^f^Percentage of those who underwent magnetic resonance imaging.

^g^Percentage of those who underwent electroencephalogram.

^h^Active rehabilitation at a specialized neurorehabilitation center, geriatric hospital, or outpatient rehabilitation center.

**Table 3.** Clinical Symptoms and Signs During Acute Phase and Follow-up in Patients With Tickborne Encephalitis Vaccine Failure in Stockholm County, Sweden, 2006–2015

Symptoms and Signs	0–2 Weeks Acute Phase	2 Weeks–3 Months	3–6 Months	6–12 Months
	(n = 53)	(n = 42)	(n = 15)	(n = 18)
	(%)	(%)	(%)	(%)
Headache	43 (81)	9 (21)	2 (13)	2 (11)
Sensitivity to light/sound	8 (15)	1 (2)	2 (13)	1 (6)
Radiculitis	17 (32)	6 (14)	1 (7)	0
Cranial nerve affection	19 (36)	3 (7)	3 (20)	1 (6)
Spinal nerve paresis	18 (34)	17 (40)	8 (53)	8 (44)
Dysphasia	30 (57)	3 (7)	2 (13)	2 (11)
Apraxia	11 (21)	1 (2)	0	0
Ataxia	38 (72)	26 (62)	9 (60)	4 (22)
Epilepsy	8 (15)	1 (2)	0	0
Concentration disability	43 (81)	22 (52)	8 (53)	7 (39)
Memory disturbance	32 (60)	17 (40)	8 (53)	6 (33)
Fatigue	52 (98)	33 (79)	13 (87)	8 (44)
Emotional lability	12 (23)	17 (40)	8 (53)	6 (33)
**Total**	**53 (100)**	**33 (79)**	**13 (87)**	**8 (44)**

### Magnetic Resonance Imaging and Electroencephalogram

Magnetic resonance imaging was performed in 25 (47%) patients, and 9 of them (36%) had pathological findings (Table 2). The affected areas were the diencephalon, with involvement of the thalamus and the basal ganglia in 7 patients and the brainstem with changes in pons, medulla oblongata, and midbrain in 4 other individuals. Electroencephalogram was performed in 23 (43%) patients with moderate or severe disease, of whom 20 (87%) showed diffuse slow activity consistent with encephalitis and 2 showed epileptic activity (Table 2).

### Clinical Features Over Time and Long-term Sequelae

Symptoms such as headache, sensitivity to light and sound, radiculitis, cranial nerve affection, and signs of dysphasia and apraxia were essentially related to the acute phase of the disease, whereas spinal paresis, to a larger extent, remained over time. Other sequelae that remained at follow-up during the first year were mainly cognitive disabilities (Table 3).

Twenty-three (43%) patients received professional rehabilitation at a specialized neurorehabilitation center, geriatric hospital, or outpatient rehabilitation clinic. The outcome was fatal in 3 patients (6%) who presented with brainstem encephalitis, tetraplegia, and bulbar symptoms, respectively. All of the fatal cases were aged >60 years and had underlying conditions such as chronic kidney failure stage 5 and ischemic heart disease, rheumatoid arthritis with methotrexate and corticosteroid treatment, and Hodgkin lymphoma with secondary immunoglobulin deficiency.

## DISCUSSION

In this retrospective study, of 1004 cases, we identified 53 patients with TBE vaccine failure in Stockholm County during a 10-year interval. The majority of patients were aged >50 years (81%) and had a monophasic course of infection (77%), which is in accordance with previous reports on vaccine failures [19, 31]. The vast majority of patients had severe or moderate meningoencephalitis, and the mortality rate was 6%, which is higher than previously reported [6, 8, 9]. Furthermore, 68% became ill following 3 or 4 doses of vaccine, but not 1 case of TBE was seen in anyone aged >60 years who had received an extra priming dose.

Our study represents the largest number of clearly defined cases of vaccine failure. In all, vaccine failures constituted 5% of all TBE cases in Stockholm County during the period studied. A high proportion of vaccine failures has also been described in Austria and Switzerland [9, 17]. Most likely such a high proportion of vaccine failure is the result of high vaccine coverage in a population at risk for TBE [13, 17, 32]. The definition of vaccine failure will also influence the result as well as the prevalence of infected ticks and the risk behavior of the population.

In accordance with other studies of TBE patients, including those with vaccine failures, the majority of patients in our study were male and of older age [17–19]. Focal neurological signs were frequent, and as many as one third of the patients presented with radiculitis; this is a proportion that we have not been able to confirm in other studies. Coinfection with borreliosis might be an explanation, but no case of radiculitis with a double infection was identified. Possibly this is the result of missed diagnostics, since testing for borreliosis was only performed if the clinicians suspected a double infection.

Although this study was not designed to evaluate long-term sequelae, information regarding residual symptoms was collected whenever possible. Cognitive impairment and spinal paresis were the most common permanent sequelae. Overall, 44% of patients followed for 6 months had objective or subjective sequelae. This high proportion is potentially due to selection bias, since repeated follow-up visits are likely to be justified for patients who report sequelae.

Our data are consistent with those from previous reports that have indicated a severe course of disease in the case of vaccine failures [20]. Data from unvaccinated cases of TBE have shown that a higher age correlates with an increased risk of severe disease [31, 36, 37]. The high median age in our cohort of vaccine failures might explain the severity of their disease. On the contrary, a recent study [19] has suggested a non–age-related increased risk of severe TBE disease in patients with vaccine failure. In that study, 13/39 patients had not adhered to the recommended vaccine schedule and were only infrequently vaccinated. All vaccinated patients’ serological responses differed from those of nonvaccinated patients, indicating a persistent immune memory regardless of time since the last vaccination. Unfortunately, the study by Lotrič-Furlan et al did not give information concerning underlying immunosuppression [19]. In general, data concerning the effect of comorbidities and immunosuppression on the outcome of TBE are scarce. In our study, half of the patients had other comorbidities, and 26% had diseases that involved the immune system, such as rheumatic diseases and malignancies. Eight patients (15%) were on immunosuppressive treatment. From the patients’ medical records, we could only confirm that 5 had been vaccinated during ongoing immunosuppressive treatment. Although immunocompromised patients represent a heterogenic group it has been shown that immunosuppressive drugs have a negative effect on the vaccine response [16].

The majority of patients (68%) with vaccine failures had previously received 3 or 4 vaccine doses. In those aged 50–59 years, 8 cases had received 3 doses, thus becoming ill in the interval of 3 years waiting for the fourth dose. There was no corresponding case in those aged >60 years who received an extra priming dose, thus waiting for the fifth dose after 3 years. Eleven patients, all aged ≥60 years at the time of infection, had received as many as 5–9 doses in total, but no extra priming dose. In this group, only 1 patient was aged <50 years when the vaccination schedule was started. Vaccination studies have demonstrated an impaired vaccination response in terms of IgG titers and neutralizing antibodies with increasing age [15, 38, 39], thus, the age at time of primary vaccination and missing extra priming dose were likely disadvantages for this group regardless of repeated vaccine doses. Furthermore, it has been shown that older individuals reached the same antibody titers after 4 vaccine doses compared to younger individuals after 3 doses, with no further differences after additional doses [14]. This adds to our observation that an extra priming vaccine dose seems to improve protection among the elderly. In 2010, notification data identifying TBE cases with vaccine failure resulted in a change to the Swedish vaccination recommendations, adding an extra dose in the priming vaccine schedule to all individuals aged >60 years. In our cohort, there were no cases of vaccine failures in patients aged ≥60 years who had received that recommended extra priming dose. Three individuals aged >60 years who did not receive an extra priming dose became ill during a 5-year interval waiting for a booster dose, thus these individuals might have benefited from an earlier booster vaccine dose at 3 years, as recommended by the vaccine manufacturers, instead of every 5 years according to the Swedish recommendations. On the other hand, neither of these individuals had received the extra priming dose; from an immunological point of view, we consider the importance of proper priming, that is, an extra dose in the first year, to be more important than booster doses [40]. Based on our findings, the Swedish recommendation of booster doses every 5 years, regardless of age, seems to be valid.

The main strength of our study is the completeness of the notification data as well as careful documentation of vaccine doses and clinical data. The retrospective study design is a limitation, given that data might be missing from the medical charts. Thus, it is possible that some signs and symptoms may be more frequent than reported. Furthermore, in case of vaccine failures, the antibody responses generally deviated from the responses in unvaccinated patients with TBE, with an early rise in IgG titers and neutralizing antibodies and late development and detection of IgM [18, 26], suggesting that cases of vaccine failures might have been overlooked and not properly diagnosed with TBE. Patients with clinical meningitis are, however, often hospitalized, and we believe there is an increased awareness of TBE despite vaccination since the Swedish publication by Andersson et al [18]. Patients with milder symptoms who did not seek healthcare were not reported, but this should not influence the findings in notified cases and, thus, should be of less importance to the results. Another limitation would be the lack of information concerning the manufacturer of the vaccine that the patients received, which was not always documented. From 2005, the regional procurement agency chose FSME-Immune; consequently, we assume that the majority of the patients received FSME-Immune, even though private vaccination clinics might have also used Encepur.

## CONCLUSIONS

To better prevent TBE in individuals aged >50 years in whom morbidity and mortality are striking, we suggest that an extra priming vaccine dose be implemented from age 50 years as has been successfully done for those aged 60 years in Sweden. This study also underlines the importance of correct data regarding notifiable diseases, including number of vaccine doses and vaccination dates. Future studies on adapted vaccination schedules in older and immunosuppressed individuals are warranted. Finally, the question of whether the clinical picture for TBE patients with vaccine failures is different from that of unvaccinated patients remains to be investigated.

## Notes

***Author contributions.*** H. H. A., L. L., S. V., M. I., and S. G.-R. conceived and designed the study. K. H. and M. I. collected all data. K. H., A. R., S. G.-R., M. I., and H. H. A. were responsible for analysis and interpretation of the data. A. R. and K. H. drafted the manuscript. M. I. and S. V. advised on methodical issues and interpretation. H. H. A., S. G.-R., L. L., S. V., and M. I. revised the manuscript critically. All authors approved the submitted version of the manuscript.

***Acknowledgments.*** The authors thank Tomas Wahl, Department of Communicable Disease Control and Prevention, Stockholm County, Sweden.

***Financial support.*** This work was supported by the Marianne and Marcus Wallenberg Foundation (S. G.-R.); the Research Committee of Orebro lan (grant number OLL-768871 to A. R.]; and the Hennes Kungliga Höghet Kronprinsessan Lovisas förening för barnasjukvård/ Stiftelsen Axel Tielmans Minnesfond.

***Potential conflicts of interest.*** All authors: No reported conflicts of interest. All authors have submitted the ICMJE Form for Disclosure of Potential Conflicts of Interest. Conflicts that the editors consider relevant to the content of the manuscript have been disclosed.
